# Machine learning techniques to predict the risk of developing diabetic nephropathy: a literature review

**DOI:** 10.1007/s40200-023-01357-4

**Published:** 2023-12-05

**Authors:** F. Mesquita, J. Bernardino, J. Henriques, JF. Raposo, RT. Ribeiro, S. Paredes

**Affiliations:** 1 Polytechnic Institute of Coimbra, Coimbra Institute of Engineering, Rua Pedro Nunes - Quinta da Nora, 3030-199 Coimbra, Portugal; 2 Center for Informatics and Systems of University of Coimbra, University of Coimbra, Pólo II, 3030-290 Coimbra, Portugal; 3 Education and Research Center, APDP Diabetes Portugal, Rua Do Salitre 118-120, 1250-203 Lisbon, Portugal

**Keywords:** Diabetic nephropathy, Kidney disease, Clinical data, Risk prediction, Machine learning

## Abstract

**Purpose:**

Diabetes is a major public health challenge with widespread prevalence, often leading to complications such as Diabetic Nephropathy (DN)—a chronic condition that progressively impairs kidney function. In this context, it is important to evaluate if Machine learning models can exploit the inherent temporal factor in clinical data to predict the risk of developing DN faster and more accurately than current clinical models.

**Methods:**

Three different databases were used for this literature review: Scopus, Web of Science, and PubMed. Only articles written in English and published between January 2015 and December 2022 were included.

**Results:**

We included 11 studies, from which we discuss a number of algorithms capable of extracting knowledge from clinical data, incorporating dynamic aspects in patient assessment, and exploring their evolution over time. We also present a comparison of the different approaches, their performance, advantages, disadvantages, interpretation, and the value that the time factor can bring to a more successful prediction of diabetic nephropathy.

**Conclusion:**

Our analysis showed that some studies ignored the temporal factor, while others partially exploited it. Greater use of the temporal aspect inherent in Electronic Health Records (EHR) data, together with the integration of omics data, could lead to the development of more reliable and powerful predictive models.

## Introduction

The widespread prevalence of diabetes is still a major public health challenge, with a significant impact on people's quality of life and an increase in mortality. Between 1980 and 2014, the number of people with diabetes increased almost fourfold, from 108 to 422 million, according to the World Health Organization [1]. In the European scenario, 6.2% of adults had diabetes in 2019. Cyprus, Portugal, and Germany were the countries with the highest levels, around 9% or more [2]. In addition, the metabolic control needed to delay diabetes complications is not achieved by the majority of patients. As a result, diabetes can cause many complications, including eye problems (retinopathy), nerve damage (neuropathy), and kidney problems (nephropathy) [3].

Diabetic Nephropathy (DN) is a chronic disease in which the function of the kidneys deteriorates, reducing their ability to eliminate wastes and toxins from the bloodstream and affecting the water balance in the body. DN is considered a progressive disease that usually gets worse over time until the kidneys can no longer function on their own, which is known as end-stage renal disease (ESRD) [4]. It is a disease that is usually considered irreversible although it has been observed that with long-term normalization of the diabetic environment, the architecture of the kidney can undergo significant remodelling and the lesions associated with diabetic nephropathy can be reversed [4]. In developed countries, half of all ESRD cases are due to DN, and the cost of treating ESRD patients is very high [5].

Digitalization has allowed hospitals to store the complete history of patient appointments in a database, resulting in the availability of EHRs. These data are longitudinal because they are collected over time and include multiple patient records at different points in time. Due to the progressive nature of many diseases, a longitudinal approach is usually required to fully assess their development and impact [6]. Given the chronic and long-term nature of diseases such as DN, it is crucial to consider the temporal dimension of patient data and not overlook its importance [7]. The timely implementation of a DN risk assessment may delay or even prevent its progression, which would certainly reduce the number of people with ESRD [8].

The dream of machines that can one day be self-learning without explicit programming is an old one [9]. Machine learning (ML) has its roots in the Artificial Intelligence (AI) movement of the 1950s, with a strong emphasis on practical goals and applications, focusing on tasks such as prediction and optimization [10]. In very simple terms, ML uses various algorithms to learn the patterns and relationships present in a dataset and ultimately predict an outcome. We are now experiencing a major and rapid transformation, brought about by significant advances in ML, which is exponentially increasing automation in many areas of society [11].

ML applied to medicine has great potential to support diagnosis by using a significant amount of patient data and processing it in a fast and intelligent way, helping physicians to make more informed decisions [12]. In fact, ML algorithms can potentially play a crucial role in a faster and more reliable way to diagnose complications associated with diabetes such as DN [13]. The application of ML techniques to analyze EHR data can provide valuable insights and enable the development of ML models that can predict the risk of developing DN or progressing to higher stages, aiding physicians in the diagnosis and ultimately improving the quality of healthcare [14, 15].

There are many studies done on the use of ML to identify cases of diabetic nephropathy. However, the focus of this research is to identify and study the approaches used on clinical EHR data collected over a period of time and the corresponding risk prediction of developing diabetic nephropathy.

This work aims to answer the following research question:

RQ: What are the most effective machine learning techniques used to construct a model that uses the temporal information in diabetic patients' EHR data to predict the development of DN or progression to higher stages?

This literature review was done in a systematic way to ensure that the results are transparent and reproducible, minimizing the bias that would result from the specific choice of studies (cherry-picking) [16].

The main contributions of this work are the following:

- We present and compare different temporal approaches used in clinical data to develop a predictive model that can accurately identify the risk of developing DN or progressing to higher stages in the future. By providing a comprehensive overview of these approaches, we aim to encourage the development of effective predictive models that can help physicians improve patient outcomes.
- We contribute to the understanding of the impact that the temporal factor can have on the prediction of DN by reviewing and comparing static and dynamic approaches.
- We identify the limitations of static and dynamic approaches and highlight the need for further research to improve the accuracy of risk prediction.
- We show that it is already possible to see that the integration of omics data can potentially improve the results and increase the credibility of predicting DN risk.

The remainder of this paper is organized as follows. Section II describes the methodology used to select the articles to be reviewed. Section III presents the results obtained. A discussion of the main findings arising from these results is presented in Section IV. Threats to the validity of this literature review are presented in Section V, while possible future research directions are outlined in Section VI. Finally, Section VII presents the main conclusions.

## Materials and methods

Three databases were used for this literature review: Scopus, Web of Science, and PubMed. These are three of the most popular and reliable sources of scientific information [17]. Only articles written in English and published between January 2015 and December 2022 were included. The search query used was:

*“((diabetes) AND ((machine learning) OR (deep learning)) AND ((time) OR (temporal) OR (time series)) AND (predict) AND ((kidney disease) OR (nephropathy)))”.*

**Fig. 1:**
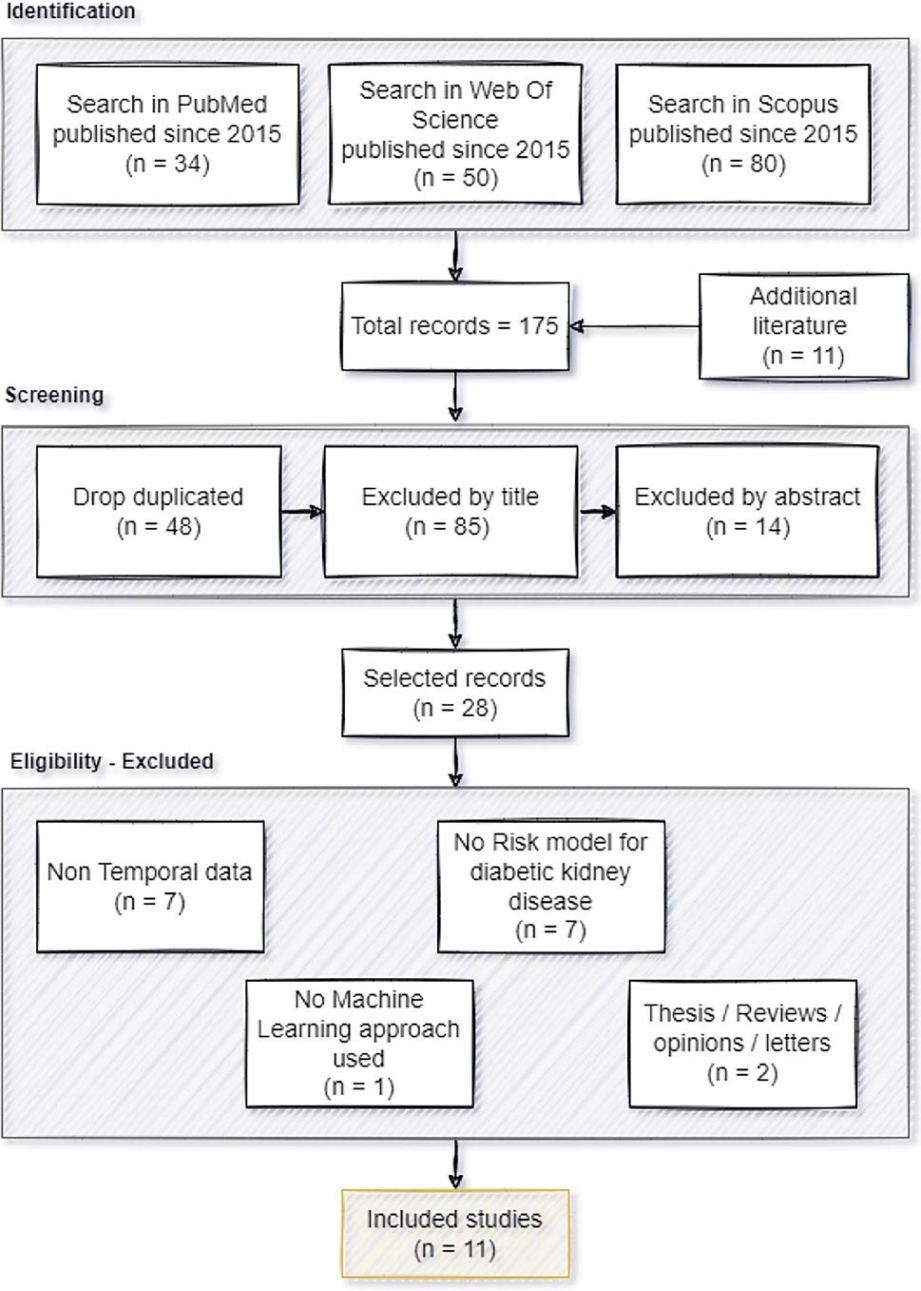
Methodology

Figure 1 describes the methodology used throughout the process. The first step (Identification) resulted in a total of 164 papers. Based on the references of some of these papers, a further 11 were identified as potentially important, resulting in 175 papers for further analysis. These 11 additional articles were referenced by papers identified in the first stage. During the screening phase, 48 duplicates were removed. In addition, 85 papers were excluded by title and 14 by abstract. These were removed because they did not relate to the intended topic; this phase reduced the original 175 to 28 papers. Of these, only 11 were eligible according to the various criteria defined. Table 1 shows a summary of the excluded articles, the criteria, and a brief explanation of the exclusion criteria.

**Table 1:** Papers excluded according to defined criteria

Papers	Criteria	Brief Explanation
[18–24]	Non-Temporal Data	Excluded papers did not include temporal data, i.e. data from patients followed up during a specific time window with information collected during that time
[25–31]	No Risk model for DN	We select articles that predict the risk of progressing or developing DN. Articles that only classify whether patients have the disease or not were excluded
[32, 33]	Thesis / Reviews / Opinions / letters	As these papers are reviews of the literature, this type of paper is not included
[34]	No ML approach	This paper has used a scoring system that defines the factors that contribute most to the development of DN. Although it is a risk model, it is not an ML approach

It should be noted that although the keyword "deep learning" was included in the search query, none of the 11 selected papers used Deep Learning (DL) techniques to solve the problem. With this in mind, we will focus only on approaches that use ML algorithms.

## Results

Following the procedure outlined in Fig. 1, 11 articles were included in this review. Artificial intelligence applied to temporal clinical data has the potential to improve the way a diabetic patient is managed according to their risk of developing DN. The different approaches are presented according to different questions: i) which features are most important, ii) what kind of ML models have been created, iii) which ones perform better, and iv) other relevant aspects. The papers selected for this review, together with a summary of their main aspects, are listed in Table 2. Looking at Table 2, we can see that most of the articles were published in the last 2–3 years, which shows a rapid growth in the application of ML to the management of diabetes-related conditions, taking advantage of the large amount of clinical data available.

**Table 2:** Summary of studies included in this review

Paper	Dataset	Pre-processing	ML Model Proposed	Performance
Singh et al. (2015) [35]	EHR data of patients in the Mount Sinai Hospital and Mount Sinai Faculty Practice Associates in New York City. From 6,435 patients, 12,337 examples were extracted	Feature selection and generation. Numerical predictors discretization into four bins based on the quartiles of the corresponding predictor and then map them into binary variables	Multitask Logistic Regression (MLTR)	≈ 68.3% AUROC for Threshold of 10% ≈ 71.2% AUROC for Threshold of 20%
Dagliati et al. (2018) [36]	943 T2DM patients in charge of the ICSM hospital and followed for more than 10 years	Data imputation with the MissForest technique and some variables were not considered because imputation errors were too high	Logistic Regression (LR)	3 years: 70.1% AUC 5 years: 73.4% AUC 7 years: 72.1% AUC
Makino et al. (2019) [37]	Dataset with 64,059 T2DM patients. From that, authors extracted structural, text, and longitudinal data	Under sampling minority class, several data transformation steps are used to summarize the last 180 days EMR records and create longitudinal data variables	Logistic Regression (LR)	AUC: 74.3%
Romero et al. (2019) [38]	Data were provided by the NHLBI, sponsor of the ACCORD trial. There were 10,251 T2DM patients from 77 clinical centers in the United States and Canada	SMOTE technique used to balance target, feature selection using the information gain metric	Random Forest	88.7% Accuracy
Sarkosh et al. (2020) [39]	Clinic of Imam Khomeini Hospital Complex (IKHC) dataset with 10,636 T2DM patients followed from 10 years (2012–2021)	Feature selection using Recursive Feature, elimination (RFECV) and RF method, imputation or drop missing values	Logistic Regression (LR)	75.5% AUC
Aminian et al. (2020) [40]	287,438 T2DM patients from Cleveland Clinic’s EHRs followed between 1998 and 2017. Two different groups were created: 2,287 patients undergoing metabolic surgery and 11,435 matched non-surgical patients	Missing data imputed using multivariate imputation by chained equations (MICE), variables with more than 25% missing values or no predictive value were removed	Random Forest	Surgical patients: 73% AUROC Nonsurgical patients: 76% AUROC
Song et al. (2020) [41]	University of Kansas Medical Center’s HERON clinical data repository with 35,779 T2DM patients	Features with less than 1% representation were removed and missing values imputed	Gradient Boosting Machine (GBM)	AUROC: 83%, 78% and 82% in predict DN in 2, 3 and 4 years, respectively
Chan et al. (2021) [42]	BioMe Biobank at the Icahn School of Medicine at Mount Sinai and the Penn Medicine Biobank data sources. Population of 1146 T2DM with both EHR data and biomarkers	Data harmonization, only variables in more than 70% participants were included, feature selection based on SHapley Additive exPlanations (SHAP) values, and missing data imputation	Random Forest	AUC: 77%
Allen et al. (2022) [43]	111,046 EHRs of T2DM patients that represents more than 700 healthcare sites from USA between 2007 and 2020	Standardization, impute missing values	Random Forest	74.8% AUROC for any DN stage, 82.3% for stage 3–5 82.1% for stage 4–5
Dong et al. (2022) [44]	Data from PLA General Hospital with 2809 T2DM patients that were followed from 2008 to 2019	Drop features with missing data > 25%, missing values imputation with RF, feature selection using RFE	LightGBM	AUC: 81.15%
Al-Sari et al. (2022) [45]	T1D cohort in Steno Diabetes Center Copenhagen (SDCC) with 537 patients with follow-up data. Later, blood molecular data with 965 features was also included	Remove high correlated features, outliers, and clinical variables with no predictive power on metabolic data Feature selection SHapley Additive exPlanations (SHAP) values	Random Forest	DN model with only clinical data: 92% of AUROC, DN model with clinical and omics: 99% AUROC

### Data sources

With the emergence and growth of available data, ML models have increased the predictive potential in a wide range of tasks in several application areas. With digitalization, all patient’s data is stored in computer databases. In fact, Electronic Health Records (EHRs) contain vital information about the patient, such as their medical history, illnesses, medications, treatment plans, allergies, and other highly relevant information. This type of data helps clinical research enormously by making it easier to access and track patient data [46]. It also allows for temporal and longitudinal analysis of the data, leading to different approaches and more accurate and correct predictive capabilities [47].

In addition to clinical variables, Omics-based biomarkers are often used. These can be defined as a molecular signature that is identified using omics data and used to predict the presence or risk of a particular disease or condition, or to monitor the response to a particular treatment. Omics can be divided into different research areas such as proteomics (proteins), transcriptomics (RNA), genomics (genes), metabolomics (metabolites), lipidomics (lipids) and epigenomics (methylated DNA) [48].

The integration of omics data with clinical data can significantly improve the ability to analyze and predict complex diseases using ML [49]. The work of Al-Sari et al. [45] is a very good example of the benefits of combining Omics data with clinical data. The performance of some of the models, which had previously been built using only clinical data, increased significantly when Omics data were included. In this case, metabolites, ketones, and sugar derivatives were used. In general, the integration of molecular data will lead to better prognostic models, as demonstrated in several works [50–53]. Despite the many benefits of integrating this type of data, there are some challenges. Sometimes, even when these data are available, they are very difficult to handle, process, analyze, and finally integrate. This requires specialized knowledge in the branches of mathematics, statistics, biology, and computer science [54].

### Feature importance

There are several factors that can lead to the onset or development of DN, such as demographic and genetic factors, clinical measurements, laboratory tests, and medical history. Most of the selected studies used different methods to understand which variables had the greatest influence on the final outcome when predicting risk. Some of these techniques were used to perform feature selection, which can potentially lead to better performance [55].

The work of Chan et al. [42] and Al-Sari et al. [45] used SHapley Additive exPlanations (SHAP) to understand how each feature contributes to the model's predictions, by estimating the amount that each variable contributes to the predicted value of an output. This allows them to ensure that they are selecting the most optimal set of variables for the task.

Recursive Feature Elimination (RFE) is an iterative method that can recursively remove the least important features from a dataset and build a model on the remaining attributes. As presented in Sarkosh et al. [39] and Dong et al. [44], this technique is very useful for selecting a subset of features that aggregates the most important features from a larger dimensional space. In both cases, a variant of this method, Recursive Feature Elimination with Cross-Validation (RFECV), is applied. A very similar approach was adopted by Makino et al. [37] and Dagliatti et al. [36] with their logistic regression (LR) stepwise feature selection method based on the Akaike information criterion (AIC). Stepwise feature selection is a method of selecting a subset of features by iteratively adding or removing variables. The AIC is a trade-off between model goodness and complexity, and measures the relative quality of a statistical model [56]. It can be used in stepwise feature selection to evaluate the performance of the model at each step and decide which feature to add or to remove. Although it appears similar to the RFE method, this technique trains on the selected subset of features at each step and can use either forward selection or backward elimination, whereas RFE trains on all features and removes the least important feature at each step.

Aminian et al. [40] computed the relative importance of each feature in the final model using AIC for the regression models and the Concordance index (C-Index) for the RF models. The C-Index is a metric that considers the temporal dependence associated with the model result and can be used to rank features by importance or even to analyze the global performance of the model.

Singh et al. [35] use a simpler and faster approach, Univariate feature selection, to identify the most relevant variables. These features were chosen through individual statistical tests with the target variable, without considering inter-feature dependencies or relationships.

Song et al. [41] adopted a slightly different approach, using the GBM classifier because it uses an embedded method of feature selection during model training.

Table shows the clinical variables that were mentioned in more than three papers as one of the most prominent variables able to give high predictive power to the model for analyzing the emergence or development of DN, and their respective meaning. Two of the reviewed articles indicated molecular data as being of high importance for the predictive model (Table 3).

**Table 3:** Most important clinical variables identified

Papers	Feature	Meaning
[42, 45], [38, 44]	eGFR or GFR	Glomerular filtration rate (GFR) measures how well the kidneys work. eGFR is an estimate, usually calculated using the Modification of Diet in Renal Disease (MDRD) equation and the Chronic Kidney Disease Epidemiology Collaboration (CKD-EPI) equation
[42, 45], [38, 44]	UAlb or Alb	Albumin levels in the blood. Low levels of this protein are called hypoalbuminemia, and high levels are known as hyperalbuminemia
[39, 45], [36, 44]	HbA1c	Glycated hemoglobin (HbA1c) measures glucose levels over the past 2 to 3 months
[39, 42], [38, 40]	UACR or ACR	Laboratory tests are used to detect proteinuria, the presence of protein (usually albumin) in the urine
[39, 44], [40, 41]	Age	In some articles, it is the age of the patient, in others it is the age at which the patient started to be followed
[39, 44], [36, 40]	BMI	Body Mass Index uses a person's height and weight to calculate an estimate of body fat

Table 4 details the three plasma biomarkers selected by Chan et al., while Table 5 shows the five molecular variables selected by Al-Sari, (2 ketones and 3 sugar derivatives).

**Table 4:** Most important omics identified by chan et al. [42]

Molecular feature	Meaning
TNFR1	Tumor necrosis factor receptor 1 is a protein found on the surface of cells that binds to TNF (tumor necrosis factor), a signaling molecule involved in inflammation and cell death [57]
TNFR2	Protein related in structure and function to the TNFR1 protein and also related to TNF, which plays a role in inflammation and cell death [57]
KIM1	Kidney injury molecule 1 is a protein produced in the kidney that is considered a biomarker of acute kidney injury and plays a role in the repair and regeneration of kidney cells [58]

**Table 5:** Most important omics identified by Al-sari et al. [45]

Molecular feature	Meaning
3,4 dihydroxybutanoic acid	Chemical compounds are found in many foods and also produced by the human body as a byproduct of some amino acids
2,4 dihydroxybutanoic acid	Also, a chemical compound like 3,4-dihydroxybutanoic acid with only small molecular differences
ribitol	It is a five-carbon sugar alcohol used as sweetener. Naturally occurring compound found in small amounts on fruit and vegetables
ribonic acid	Also found in small amounts on fruit and vegetables, but it is also a metabolic pathway intermediate and a byproduct of xylose fermentation
myo-inositol	Six-carbon cyclic sugar alcohol. A naturally occurring compound found in some foods, particularly fruits and nuts. It is also produced by the human body as a byproduct of glucose metabolism

### Risk models

This section systematizes several approaches to building a model that can predict the risk of developing diabetic nephropathy. Some approaches do not fully exploit the time factor inherent in the data (static approaches), while others manage to make better use of this factor (dynamic/temporal approaches).

### Static approaches

Dong et al. [44] used data from non-DN patients at baseline who were followed for three years. The authors then used 408 patients who remained without DN and 408 patients who developed DN after the follow-up period. This data was used to build the model, it contains all the characteristics that the patient presented at baseline and the variable to predict is whether they developed the disease after the three years of follow-up. Binary classification was performed using seven different ML classifiers: Light gradient boosting machine (LightGBM), eXtreme gradient boosting (XGBoost), Adaptive boosting (AdaBoost), Artificial Neural Networks (ANNs), Decision Tree (DT), Support Vector Machine (SVM), and Logistic Regression (LR). This binary classification predicts the presence or absence of DN within 3 years.

There are several other papers that have taken a similar approach and transformed the problem into a binary classification. Romero et al. [38] followed a similar strategy, but defined eight different time windows for all the 7 years of patient follow-up data. Each window corresponds to one year of data, except for the first two windows, which correspond to only 6 months each. The tree-based classifiers OneRule, J48, and RF were chosen for their simplicity, speed of classification, and user-friendly graphical presentation.

Dagliatti et al. [36] used a binary outcome variable but for three different time thresholds of 3, 5, and 7 years to predict the risk of DN. LR, Naïve Bayes (NB), SVM, and RF were tested.

Aminian et al. [40] used data from both surgical and non-surgical patients with T2DM. Multivariate time-to-event regression and RF models were created to predict the 10-year risk of developing DN for both patients with and without metabolic surgery.

Sarkosh et al. [39] trained an LR-based risk score in 1907 diabetic patients, of whom 763 developed DN within five years. In a binary outcome problem, the authors used multivariate LR analysis to generate risk scores and divided patients into four different groups based on their respective risk of DN: low, moderate, high, and very high.

Chan et al. [42] used the same binary outcome in a train/test set of 686 patients and a validation test of 460 patients. Using clinical data and biomarkers, the authors generated risk probabilities using the final RF model and scaled the results to a continuous score between 5 and 100. The authors named the whole system IntelKidneyX. It stratified patients as follows: low risk (46%), intermediate risk (37%) and high risk (17%) of developing DN within 5 years.

Al-Sari et al. [45] and Makino et al. [37] did almost the same as the previously cited papers, but instead of defining outcome as absence or presence, it was defined as progressor or non-progressor in the Al-Sari paper and as worsening or stable in the Makino et al. paper. Al-Sari et al. used data from 190 patients who had no progression of DN and 190 patients who had progression of DN during a mean follow-up of 5.4 years. He used the RF classifier to predict whether the patient would progress to DN during the follow-up period. On the other hand, Makino et al. extracted clinical features from longitudinal, textual, and structural data. LR models were trained using data from 15,422 stable patients (remaining DN stage 1) and 15,388 patients who experienced disease progression at some point (from DN stage 1 to DN stage 2–5).

Unlike the works presented above, Allen et al. [43] are able to predict 3 different outcomes, DN progression to any stage, DN progression to stages 3–5, and DN progression to stages 4–5. Three different models were created for each possible outcome, each predicting the risk of progression to DN over the next 5 years. RF and XGBoost were used as classifiers with a training and test set of 62,994 and 7,656, respectively.

**Fig. 2:**
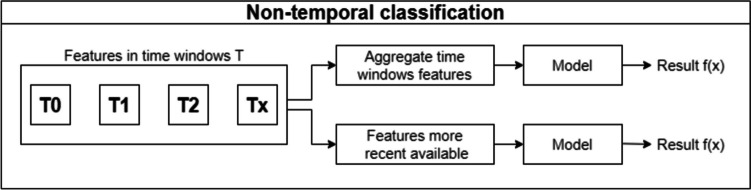
Non-temporal approaches

Figure 2 provides a general overview of the different approaches described above.

### Dynamic approaches

Different temporal approaches have been proposed to deal with EHR and provide risk prediction for DN. Within the remaining selected articles, the following approaches were used: stacked temporal, multitask temporal, discrete survival, and landmark boosting.

**Fig. 3:**
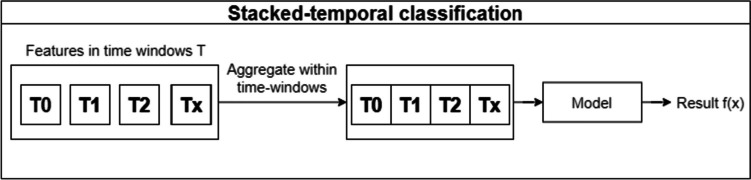
Stacked temporal approach

The stacked temporal technique was used in both Singh et al. [35] and Song et al. [41] work. It aggregates data from each time window to create a single prediction. T time windows, with F features in each, result in only one time window with T multiplied by F features. One of the disadvantages of this technique is that the larger the temporal space considered, the higher the dimensionality of the data, which can lead to a large overfitting. In Fig. 3, the physician appointments within each time window are aggregated to form a one-dimensional space, which is then fed into the model and a prediction is obtained.

**Fig. 4:**
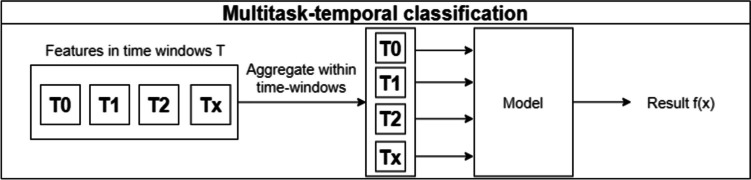
Multitask temporal approach

The multitask temporal method, introduced by Sing et al., involves predicting the outcome separately for each time window, with the requirement that each window must contain at least five physician appointments. When predicting the risk of DN for a new patient, time windows with five or more appointments are used and the final prediction is the average of the different results obtained in each time window. This stratification of the problem is shown in Fig. 4.

**Fig. 5:**
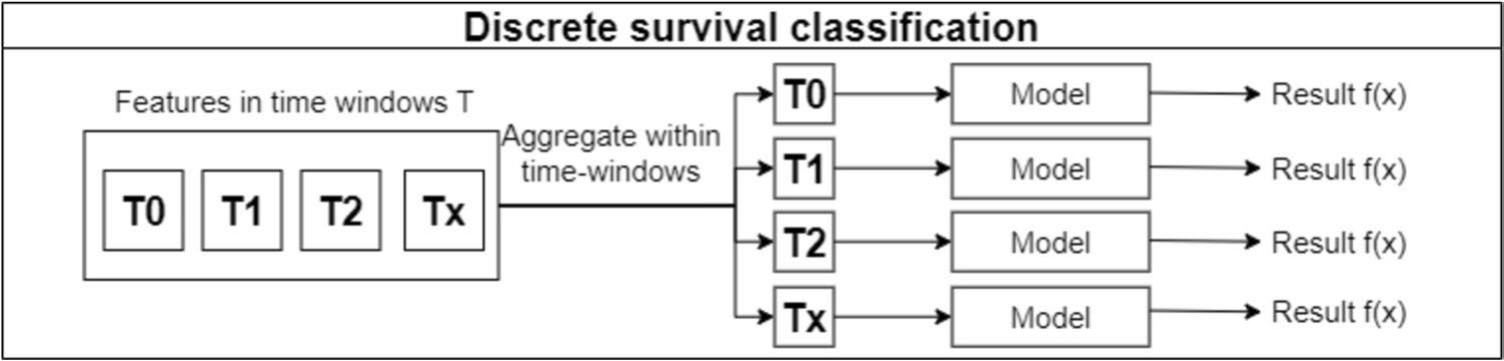
Discrete survival aproach

Discrete survival and landmark boosting are two techniques mentioned in the paper by Song et al. The first makes an individual prediction in each time window, with no overlap between windows. A disadvantage of this technique is that it assumes that there is no relationship between examples in different time windows, even if they come from the same patient. This can be seen in Fig. 5.

**Fig. 6:**
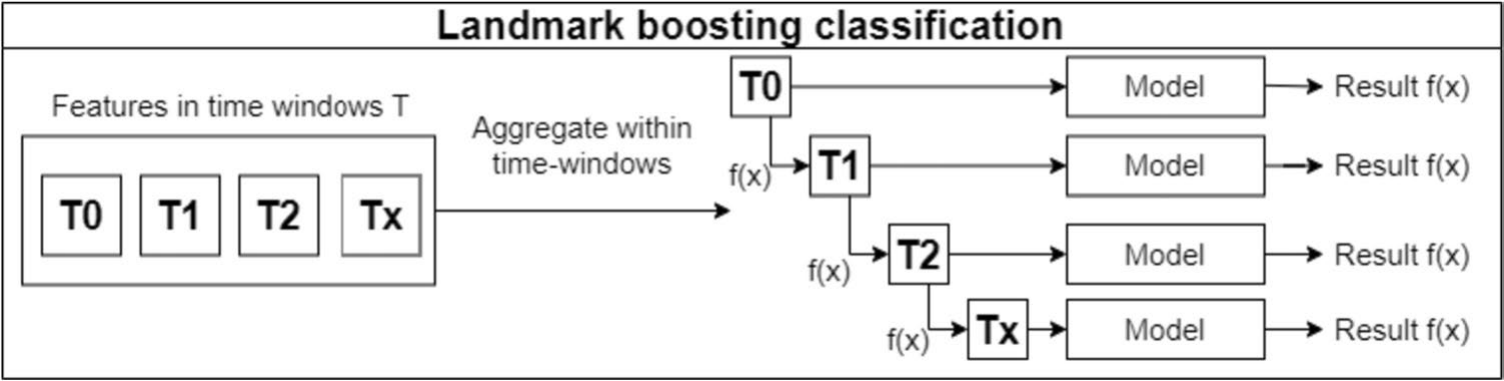
Landmark boosting classification

**Fig. 7:**
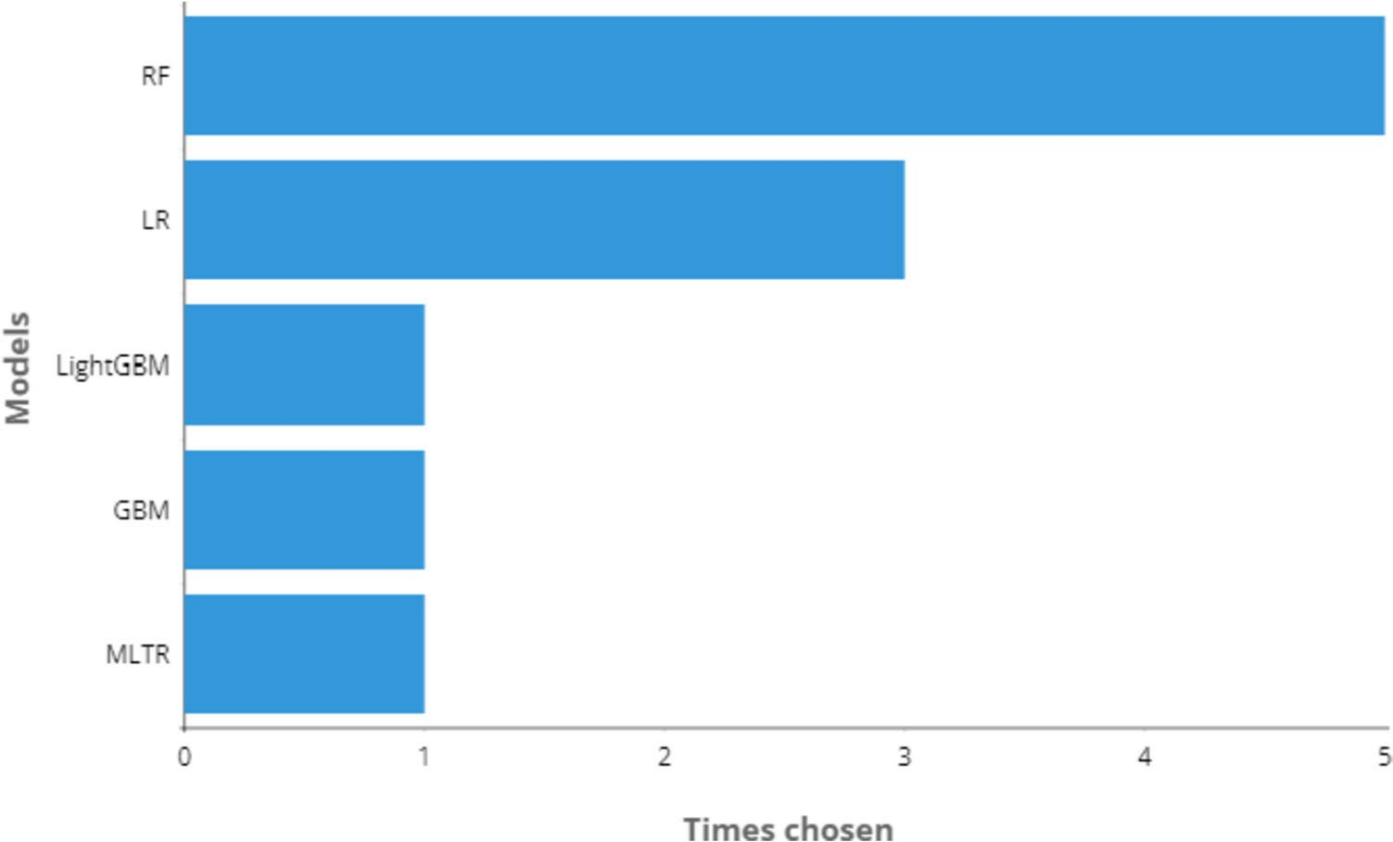
Most used ML classifiers in proposed methods

On the other hand, landmark boosting is very similar to discrete survival, but in each time window *t*, the prediction made in the previous time window *t – 1* is also considered. In effect, there is a transfer of knowledge between the time windows, making each prediction more accurate. This can be seen in the representation of the approach shown in Fig. 6, where each model receives not only the features corresponding to a time window, but also the prediction made in the previous time window (Fig. 7).

### Used models, interpretation, and performance

This section discusses the type of models most commonly used to predict the onset or development of DN. It also presents the main interpretation techniques used and a comparison of performance.

Considering the selected papers, five different classifiers were proposed: RF, LR, LightGBM, GBM, and Multi-Task Logistic Regression (MTLR). From Fig. 7, we can see that the most selected method was RF, followed by LR, and finally LightGBM, GBM, and MTLR, which were selected only once.

Performance is the most important individual factor that defines the classifier, but it is not the only aspect to consider. RF was the most used classifier because the decision trees that make it up can be interpreted and the final result can be explained [43]. It has a good classification speed and can be represented graphically [38]. However, as a whole, these methods are often difficult to interpret, especially when the number of decision trees is large. It is therefore a classifier with a good balance between speed, complexity, and interpretability. LR has also been proposed several times because it provides a clear interpretation of its coefficients, which are usually represented graphically by nomograms, concepts with which physicians are very familiar [36, 59]. GBM was chosen by Song et al. [41] because of its robustness and effectiveness in predicting DN risk, as demonstrated in previous work. In addition, it incorporates feature selection. MTLR was proposed by Singh et al. [35] because it was appropriate for the type of solution proposed in their multitask temporal methodology. It consists of a multitask learning approach where learning is performed in parallel, and tasks are related to each other [60]. In this case, there is a learning task for each time window, and this approach is used to capture the dependency between tasks.

It is possible to identify three main techniques to interpret the results generated by the predictive model: i) SHapley Additive exPlanations—SHAP values, ii) monograms, iii) decision tree visualization. SHAP values were proposed by Lundberg et al. in 2017 to analyze model predictions [61]. It calculates the importance of each feature for a given prediction, where each feature can have a positive or negative impact on that specific prediction. The contribution of features can be local (each observation) or global (set of observations). In this case, authors used local explanations to show the reasons that lead to a certain result generated by the model for a specific patient. Nomograms are graphical representations of LR models. They work like scoring systems, where each feature is assigned a certain number of points according to its value, and the result varies according to the number of points accumulated in the sum of the different features [62]. Finally, some of the articles used only tree-based models because they can be interpreted directly by visual inspection of the associated decision tree [63].

Some papers predict the onset of DN, some predict the worsening, and some authors predict the worsening for specific stages of the disease. In addition, there are papers where the result corresponds to only one specific time window, while others implement a different prediction for each time window, taking into account a certain number of years. This heterogeneity makes it difficult to compare their performance directly. Table 6 provides detailed information on each of the proposed methods.

**Table 6:** Details and performance of proposed methods

Proposed method	Time range	Outcome variable	Performance metrics
Random Forest [43]	5 Years	Multiclass (DN advance to any stage, DN advance to stage 3–5, and DN advance to stage 4–5)	Any stage—AUROC: 0.748, Sensitivity: 0.7, Specificity: 0.662 DN stage 3–5—AUROC: 0.823, Sensitivity: 0.750, Specificity: 0.739 DN stage 4–5—AUROC: 0.821, Sensitivity: 0.751, Specificity: 0.712
Random Forest [40]	10 Years	Binary target (morbidity and mortality risks)	AUC: 0.76
Random Forest [42]	5 Years	Binary	AUC: 0.77
Logistic Regression [36]	3, 5 and 7 years	Binary	3 years—Accuracy: 0.647, Sensitivity: 0.820, Specificity: 0.730 and AUC: 0.808 5 years—Accuracy: 0.693, Sensitivity: 0.750, Specificity: 0.616, AUC: 0.734 7 years—Accuracy: 0.686, Sensitivity: 0.714, Specificity: 0.643, AUC: 0.721
LightGBM [44]	3 years	Binary (DN presence or absence)	Accuracy: 0.768, Sensitivity: 0.741, Specificity: 0.797 and AUC: 0.815
Logistic Regression [37]	6 months	Binary (DN stable or aggravation)	Accuracy: 0.701 AUC: 0.743
Random Forest [45]	Non-defined	Binary (DN progression or no progression)	Accuracy: 0.96 AUC: 0.96
Random Forest [38]	8 time windows at a max of 7 years	Binary on each time window	Average Acc: 0.887
Logistic Regression [39]	5 years	Binary (DN presence or absence)	AUC: 0.758
Multitask Logistic Regression [35]	5 years with time windows of 6 months	Binary on each time window	≈ 68.3% for Threshold of 10% ≈ 71.2% for Threshold of 20%
GBM [41]	2, 3 and 4 years	Binary on each time window	2 years—AUROC: 0.830 3 years—AUROC: 0.780 4 years—AUROC: 0.820

## Discussion

To the best of our knowledge, this is the first literature review that explores works that make use of EHR data from longitudinally monitored patients to create a predictive DN risk model within a specified time frame*.* This paper can be used as a basis for further research aimed at in-depth analysis and optimizations on the use of the temporal factor. Such efforts might lead to the development of high-performance predictive models capable of taking advantage of the patient's history to anticipate the onset of diseases such as DN.

There are several approaches in the literature for handling EHR data that are collected over time and then used to build a model to predict the risk of the onset / development of diabetic nephropathy within a given time period. This is a very heterogeneous area of research, where there is no well-defined approach to achieving the previous goal. As Fletcher points out, *heterogeneity can be, and usually is, a good thing and can be beneficial* [64]. 

The main findings that have emerged from this work are as follows:

- There is very little work that takes full advantage of the time factor inherent in EHR data. The works of Sing et al. [35] and Song et al. [41] are an exception. In fact, the landmark boosting method proposed in the Song et al. paper was the approach that took more advantage of the time factor. It not only predicts the risk in each time window, but also takes into account the result produced in the previous time window. Although this approach attempts to exploit the full temporal potential of EHR data, it could still be improved, as it considers all records as independent, which is not the case since each patient has multiple records (appointments).
- Combining omics data with clinical data can help better predict the risk of DN over time, as confirmed in the work of Al-sari [45]. In the near future, this type of data will be linked to disease risk models because the information they contain is really valuable to increase the predictive power of the different risk models.
- Another important concern with clinical risk models is interpretability. Almost all of the proposed models were selected not only because of their good performance but also because they allow interpretation of the respective results.
- The vast majority of the selected articles were published recently (within the last 3 years), demonstrating the importance of studying existing clinical data (EHR) through longitudinal analyses, and the potential that these approaches can have in supporting patient follow-up and medical decision making.

Despite the great capabilities and improvements that these proposed models can potentially bring to medical care, the various papers reviewed have limitations, that are clearly stated by the authors. Some of the most commonly cited limitations are as follows:

- The patient sample was clinic-based rather than population-based, which means that the model was only tested on a particular dataset, extracted from the population of a particular hospital/clinic. Furthermore, in most studies, there is no external validation dataset, leading to great uncertainty about generalization to a wider population. Cabitza et al. [65] show how external validation is essential for building robust predictive models in medicine.
- Small data samples, too much missing data and missing important features. Models trained on a small amount of data can result in poor generalizability and lead to incorrect conclusions being drawn. Too much missing data can affect the consistency of the data across different visits by a given patient. This consistency is essential to build a model that can deal with the time factor and make a prediction. In addition, several papers have highlighted various missing demographic, clinical, and laboratory variables that may be essential to improve outcomes.
- Almost all of the selected papers assume that the examples are independent of each other, which is inaccurate because multiple records belonging to a single patient have been obtained. The ability to account for this inter-record dependency is key to unlocking the potential that may exist in the temporal value of EHR data and can lead to models with greater and better predictive ability. Considering this relationship, Song et al. [41] simulated some inter-record dependency by passing the prediction made in each time window to the prediction of the next time window.

Using the information obtained from the selected articles, we are now able to answer the proposed research question.

RQ: What are the most effective machine learning techniques used to construct a model that uses the temporal information in diabetic patients' EHR data to predict the development of DN or progression to higher stages?

The reviewed literature suggests that despite the potential of using ML techniques to fully exploit the temporal dimension of EHR data to predict the risk of developing or progressing to DN, this has not yet been fully achieved. Many of the techniques used have limited use of the temporal dimension and richness of patient records available in EHR data. Approaches that use only the values available for each patient at baseline or that use statistical operations on the data to combine aggregations of different clinical visits into a single record are valid but completely ignore the temporal potential. There are also some approaches that try to make a longitudinal study of the data, but often in a somewhat incomplete way. For example, the forecasts are separated by time windows (1 year from now, 2 years from now, etc.) and in some cases these forecasts are completely independent of each other. This completely breaks with the value of time and creates a shortcut to a result that is not very different from the first approach. The Landmark Boosting approach proposed by Song et al. was able to stand out because it creates time windows and tries to establish a correlation between these windows by predicting the disease state in the current window based on the state predicted in the previous window.

In summary, all the papers included in this review were generally able to arrive at a workable risk model for the onset or development of DN using a variety of techniques. All of them have attempted, either statically or dynamically, to make partial use of the temporal factor.

## Threats to validity

This section discusses all the potential threats to the validation of this work, and the various biases and weaknesses that could in any way jeopardize the results obtained.

This review uses only three different databases, and the search was done with only one query (although it included all relevant keywords). This may introduce a selection bias, meaning that our sample of studies may not be representative of the population studied. If more papers had been included, we would be more likely to have different approaches that could add value to the discussion and possibly change the conclusions drawn.

The heterogeneity of the studies also threatens the validity of this paper. The data differ in quantity, in time of collection, demographic, social and cultural characteristics of the patients, and in some cases even in the meaning of the dependent variable (outcome). Some of them had multiple disease outputs and were not specifically designed to predict the risk of DN. This results in different training and validation data between the different articles selected. They also do not have a standardized way of presenting the results. In addition, some papers omit important information, which can lead to inaccurate or inconsistent results and conclusions. This is commonly referred to as measurement bias.

The study provides a broad and consistent approach to models capable of creating a predictive model of DN using EHR data and their respective time factors. However, it is important to consider that these errors and biases may have altered or influenced the results obtained and the conclusions drawn from them.

## Future research directions

Given the small number of works that have been done in this area, there is a great need for future research to have a clearer perspective on the impact that temporal data analysis could have on medical support systems [66]. In the coming years, it is expected that there will be a huge growth in this type of work, as shown by the trends in the studies selected for this review. Therefore, the following future research directions can be outlined:

- Fully exploit the time factor: Developing strategies that take advantage of the time factor and the dependency between different visits for the same patient, not only to obtain more data, but also to allow the algorithm to access and consider the data as a healthcare team would normally do.
- ML with omics data: Further and better research into the impact that omics data can have on DN prediction by ML models should be explored so that it is possible to measure the impact of the respective integration. With the advent of modern biotechnologies and the great potential of ML, there is a great opportunity to bring together ML and omics data to significantly improve current systems [67].
- Apply Deep Learning (DL) techniques: Future research should focus on addressing the temporal nature of EHR data, as most traditional machine learning models are limited in their ability to handle this factor. One promising approach is the use of DL algorithms, which are well suited for detecting hidden patterns in large volumes of data and have greater flexibility and generalizability [68]. Therefore, the application of state-of-the-art DL techniques in future studies could potentially unlock the full temporal potential of EHR data and significantly improve predictive ability.

## Conclusion

This review focused on approaches that can use longitudinal data (EHR) to create ML models capable of predicting the risk of onset or development of DN. The findings suggest that the time factor inherent in the data has a clear potential to create a better predictor of DN risk. In addition, the combination of clinical and omics data can help us to achieve better results with greater credibility and generalizability. Furthermore, it is possible to test the concern of the authors of the different papers to create interpretable models whose results can be easily explained and understood by healthcare professionals.

It is important to emphasize that the studies varied in population, type and amount of data, outcome, and even purpose of the study, which may lead to limitations in the findings of this review. Further research is needed to address these limitations and to monitor how this area of temporal analysis of longitudinal data develops in the coming years.

Currently, there are only a few studies that have partially used the temporal information from EHRs to improve the accuracy of predictive ML models. However, we believe that using these temporal data will have a significant impact, especially in the detection of chronic diseases that take a long time to develop symptoms. Physicians use a patient's medical history to diagnose such diseases, and it is important for ML models to do the same. Therefore, incorporating temporal data from EHRs into ML risk prediction models has the potential to be a valuable support tool in healthcare, particularly in the diagnosis and management of chronic diseases, such as DN.
